# IT’S MY BUDDY: EXPLORING THE PERCEPTIONS OF PEOPLE WITH DEMENTIA ABOUT THE SOCIAL ROBOT PARO IN A HOSPITAL SETTING

**DOI:** 10.1093/geroni/igz038.1206

**Published:** 2019-11-08

**Authors:** Lillian Hung, Habib Chaudhury

**Affiliations:** 1 Simon Fraser University, Vancouver, British Columbia, Canada; 2 Simon Fraser University, Vancouver, Canada

## Abstract

New technology such as social robots opens up new opportunities in hospital settings. PARO, a robotic pet seal, was designed to provide emotional and social support for older people with dementia. This project aims to explore the perceptions of persons with dementia about PARO’s role in a hospital setting. Video-ethnographic methods were applied. We had conversational interviews with and video observations of 10 older people with dementia in the geriatric unit of a large Canadian hospital. Also, semi-structured interviews and two focus groups were conducted with 10 staff members in the local unit to gain contextual information. Thematic analysis yielded three substantive themes: (a) “it’s like a buddy”—the robot helps people with dementia uphold a sense of self in the world; (b) “it’s a conversation piece”—the baby seal facilitates social connection; and (c) “it’s all about love”—PARO transforms and humanizes the clinical setting. Our findings help provide a better understanding of the direct perspectives of patients with dementia on the use of social robots. Instead of substituting human contact, the social robot complements emotional care and supports our fundamental human need for love.

